# Development of New Strategies Using Extracellular Vesicles Loaded with Exogenous Nucleic Acid

**DOI:** 10.3390/pharmaceutics12080705

**Published:** 2020-07-26

**Authors:** Nicola Salvatore Orefice

**Affiliations:** 1 Department of Medicine, University of Wisconsin-Madison, Madison, WI 53705, USA; nicolaorefice819@gmail.com or nsorefice@medicine.wisc.edu; Tel.: +1-608-262-21-89; 2 Waisman Center, University of Wisconsin-Madison, Madison, WI 53705, USA

**Keywords:** gene delivery, plasmid vector, adeno-associated virus, exosomes, DNA delivery, viral vectors, extracellular vesicles, DNA loading

## Abstract

Gene therapy is a therapeutic strategy of delivering foreign genetic material (encoding for an important protein) into a patient’s target cell to replace a defective gene. Nucleic acids are embedded within the adeno-associated virus (AAVs) vectors; however, preexisting immunity to AAVs remains a significant concern that impairs their clinical application. Extracellular vesicles (EVs) hold great potential for therapeutic applications as vectors of nucleic acids due to their endogenous intercellular communication functions through their cargo delivery, including lipids and proteins. So far, small RNAs (siRNA and micro (mi)RNA) have been mainly loaded into EVs to treat several diseases, but the potential use of EVs to load and deliver exogenous plasmid DNA has not been thoroughly described. This review provides a comprehensive overview of the principal methodologies currently employed to load foreign genetic material into EVs, highlighting the need to find the most effective strategies for their successful clinical translation.

## 1. Introduction

Nucleic acid-based therapies are rapidly evolving in preclinical and clinical trials for several genetic diseases [1,2,3]. Viral vectors have been the primary carriers of nucleic acids used in gene therapy; due to their high transduction efficiency and absence of integration into the host genome, adenovirus (AV) vectors initially offered the promise of highly efficient and therapeutic in vivo gene delivery [4,5,6]. However, several issues brought to light still need to be addressed to achieve better long-term patient outcomes. The high immune response remains a serious concern; indeed, a high prevalence of anti-AV vector immunity in the human population and variable expression of receptor proteins on target cells have arisen during preclinical and clinical studies [7,8,9,10,11,12]. Furthermore, the tendency of AV vectors to be hijacked in the liver after systemic administration hampers efficient transgene transduction, causing hepatotoxicity and death [13,14,15,16,17,18]. So far, even though AVs continue being developed for multiple clinical interventions, including anticancer therapeutics, numerous vaccine efforts, and neurological disorders, many researchers’ focuses have shifted toward the development of novel viral vectors that would combine low potential clinical immunogenicity and genotoxic effects with highly efficient deliveries. Adeno-associated virus (AAV) vectors are currently the most commonly used viral vectors at transducing dividing and nondividing cells in gene therapy [19,20]. AAV vectors encompass a linear single-stranded DNA (ssDNA) genome of approximately 4.7 kilobases (kb); furthermore, these viral vectors contain two open reading frames encoding the nonstructural Rep (replication) and structural Cap (capsid) proteins, which are exchanged with the exogenous DNA of choice [21]. Once delivered, the ssDNA genome is converted into double-stranded DNA (dsDNA) by the host cell. This conversion process is carried out by two cis-acting nucleotide inverted terminal repeats (ITRs) consisting of 145 nucleotides in length, allowing the replication, packaging, and integration of the viral genome [21]. Based on some alternative AAV serotypes or designer mutants of the AAV capsid, a new generation of recombinant AAV (rAAV) [22,23], which lacks viral DNA, have recently been engineered able to pass through the cell membrane, where they can ultimately convey and deliver their cargos into the nucleus of a cell. So far, the Food and Drug Administration (FDA) has approved AAV biologicals for the treatments of spinal muscular atrophy [24,25] and inherited blindness (Leber’s congenital amaurosis) [26]. Other clinical trials are ongoing to assess the AVV therapeutic efficacy for other diseases [27,28,29]; however, likewise to AV vectors, recurrent symptoms of the autoimmune response encountered undergoing gene transfers with AAV vectors have been reported [30,31,32]. Early studies performed in preclinical models of diseases [33,34] did not reveal that the immune response could be triggered by the AAV vectors and represent a significant barrier for sustained gene expression. Generally, the mild immune response observed during preclinical studies was considered transient to affect sustained transduction negatively; this assumption probably stemmed from an inappropriate animal model that could be deficient in transgene products. However, so far, we still know very little about the consequences of these interactions triggered by the immune response against viral vectors in the clinical setting, particularly how the innate immune system to AAV affects adaptive responses to the recombinant vector. Further, it should be borne in mind that, to achieve a therapeutic concentration, the number of required injections of ever-higher adeno-associated virus (AAV) doses can cause toxicities by altering every clinical trial. Despite significant progress in the understanding of AAV biology and the development of efficient AAV vectors, this additional immunotoxicity urges further studies, including animal model-specific transgenes, addressed to avoid the likelihood of an immune response due to AAV treatments over time. Other important AAV-associated limitations, like their small DNA packaging size [35,36,37], the complications of finding the optimal tropism [38], and the gene expression-related slow onset [39], still represent issues and hurdles that impede their broad clinical adoptions in other areas. Driven by these limitations, an alternative and efficient loading and delivery system of exogenous nucleic acid remains an ongoing challenge. In recent years, the functions of extracellular vesicles (EVs) have sparked interest in a new model of introducing foreign genetic materials [39]. EVs are endogenous delivery systems with a diameter range of ~50 nm to 1 µm [40]. EVs are formed in endosomal compartments and secreted after fusion with the plasma membrane by most cell types [41,42]. They can mediate and transmit a variety of intercellular signaling molecules packaging biological cargo, including nucleic acids, small RNAs (sRNAs), proteins, and lipids altering the gene expression, proliferation, and differentiation of recipient cells during physiological and pathological conditions [43]. Although the classification of EVs is continuously evolving [40], they generally are classified on their biogenesis and release pathways, such as exosomes (Exo) (~40 to 160-nm in diameter) [44,45]; ectosomes [46]; or shedding microvesicles (SMVs), apoptotic blebs (ABs) (1 to 5-mm in diameter) [47], and other EVs subsets [46], generating a heterogeneous group of components able to redistribute their biological cargo into the entire organism. Moreover, several groups have reported the presence of DNA species, including ssDNA, dsDNA, and mitochondrial (mt) DNA encompassed in EVs from various sources [48,49,50]. The ability of EVs to carry genetic material mark their potential role in the transfer of exogenous genetic materials into the human genome [41]; additionally, EVs are used to deliver their cargo, crossing biological barriers, such as the blood-brain barrier (BBB) [51]. Several published studies report that plasmid RNA successfully loaded into EVs [52]; however, the potential use of EVs to load plasmid DNA for delivery applications remains scarcely described. The present review gathers the main methodologies currently employed to load foreign genetic material into EVs, the advantages and disadvantages associated with each methodology, and raises questions still unanswered, highlighting the importance of further explorations to optimize the loading strategies in these endogenous vectors as a new tool of the delivery system of foreign genetic materials.

## 2. Adeno-Associated Virus (AAV)-Associated Drawbacks Still Represent a Significant Restriction to Their Full Employment in Human Therapy

### 2.1. Circumnavigate the Capsid-Neutralizing Antibodies

The limitations of AAV vectors have a significant impact on their complete clinical translation into human diseases. First, the time-consuming conversion of single-stranded to double-stranded AAV genomes can delay the onset of transgene expressions [53]. The viral capsid is composed of three proteins: VP1, VP2, and VP3, in which VP2 and VP3 are shortened versions of VP1. Thus, the capsid proteins and transgene products constitute the only immunological antigens. However, since viral capsids are derived from wild-type AAV, AAV vectors can be impacted by preexisting adaptive immune responses, capsid neutralizing antibodies (Nabs), making the viral capsid the trigger of the immune response in several subjects undergoing AAV gene transfer [54]. An in vitro method was recently published to quantify AAV cell-binding inhibition by qPCR [55]; however, several hindrances associated with this assay have been reported [56]. Several strategies have been addressed to circumnavigate the capsid Nabs; the in vivo administration of a proteasome inhibitor (PI), bortezomib, has revealed a significantly reduced Nabs trigger against AAV vectors [57]. To avoid the interaction of AAV vectors with Nabs into the blood, the temporary removal of the anti-AAV capsid antibodies by a saline flush of the portal vein [58] has proven to minimize the inhibitory effects of anti-AAV antibodies and improve transductions. Finally, a nonimmune suppressive method to promote AAV administration in an individual with preexisting Nabs to viral vectors consisted of the coadministration of empty capsids that lack vector genomes in excess to saturate Nabs-binding sites [59]. However, all these efforts have been weakened by standardized protocols to assess in vitro Nabs assays that are still not available, making it challenging for a thorough investigation and to compare outcomes come from different research groups.

### 2.2. The Limited Packaging Capacity of Adeno-Associated Viruses

The other main hindrance associated with AAV vectors is their limited genome packaging capacity (~4.5 kb). For the packaging of foreign DNA molecules into AAV capsids (WT-AAV genome or any transgene cassette), the DNA sequence must be flanked by the cis-acting ITRs. These represent the packaging signals and allow the amplification of the DNA genome by a self-primed replication mechanism. The replication products are DNA genomes of positive and negative polarities packaged into the AAV virions with equal frequencies [60]. Efforts to expand the AAV vectors’ packaging capacities like the reassembly of abundant proteins in the cells (e.g., protein trans-splicing) [38,60] have been pursued; however, these still occur with lowered efficiency and a clear upper packaging threshold at 5.2 kb. For instance, as reported by Pryadkina et al. [61], the dysferlin cDNA at the exons 28/29 junction cloning can be truncated into two partially overlapping fragments separately packaged into two different AAV capsids. Even though the overlap region strategy is the simplest, it still requires extensive preclinical optimization steps to determine the most efficient overlap sequence. An additional strategy gene to express oversized plasmid DNA—in particular, employed for the expression of a mini-dystrophin—involves the miniaturization of large genes. In this strategy, significant portions of the coding sequence are deleted, leaving only the essential domains, which are then packaged into a single capsid [49]. The mechanisms that regulate the trafficking of AAV vectors into the nucleus are still not fully understood [62]; it has been deemed that these vectors enter cells through receptor-mediated endocytosis, and internalized virions escape from endosomal degradation by a low pH-dependent process. Since the capsid protein is thought to be an essential element in the intracellular trafficking of AAVs, the novel avenues are focused on engineering AAV vectors to bypass the limited packaging capacity (e.g., by oversized AAV vector genomes) [63,64] and achieve a higher transduction efficiency and specificity for relevant target tissues. However, so far, each effort to overcome these limitations still suffers from low efficiency of transfections, resulting in decreased gene expression levels.

## 3. Exosomes: Natural Shuttles to Deliver Exogenous Acids Nuclei Bypassing the AAV-Associated Drawbacks

The study on exosomes is continually growing, to yield valuable information regarding their intrinsic properties in regulating complex intracellular pathways, including those underlying neurological disorders [65]. In contrast to AAV vectors, the exosomes administration ensures the functional delivery of their cargo with minimal interference from the immune system [66] and has proven to be safe and well-tolerated. Moreover, as discussed later in this review (Section 8), the lipid and protein composition of exosomes can be used to assemble new methodologies to enhance DNA loading. An important aspect to highlight consists of the roles of exosomes to mediate the spread of pathological proteins involved in neurodegenerative diseases [67]. As a tool for inter-neuronal communication, exosomes can contribute to local synaptic plasticity and allow communication within different regions across the entire brain, influencing distant neuronal networks. This could provide a new understanding of the local propagation of neurodegenerative disease in the brain. The aggregation and deposition of misfolded proteins in defined neuroanatomical locations is a common feature of several neurological disorders [68]. As the diseases progress, the misfolded proteins spread along distinct pathways, suggesting that the pathological process may involve the movement of misfolded proteins from one side of the brain to the other [68]. Exosomes also contain misfolded and aggregated forms of neurodegenerative disease-associated proteins like Alzheimer’s disease (AD), Parkinson’s disease (PD), and amyotrophic lateral sclerosis (ALS), which have been found in the cerebral spinal fluid (CSF) and the blood of patients affected by these disorders [69]. Given that exosomes shuttle from the endoplasmic reticulum (ER) [70], in which the protein folding process occurs [71], to reach their final destination, one might hypothesize that exosomes remove misfolded proteins aggregated inside the ER to maintain cellular homeostasis.

However, the most important questions still need to have answers: How and where are misfolded proteins packaged into exosomes, and how can exosomes be recruited by recipient cells? Continued research of the complex architecture of exosomes will provide insights into how these vesicles may be therapeutically targeted in the future.

### 3.1. Heterogeneity Effects: A Complexity that Conditions the Isolation, Characterization, and Functions of Exosomes

Heterogeneity is a critical aspect in the exosomes field, given that it represents an underlying factor that has an impact on current and novel exosomes isolation techniques. Exosomes are membrane-bound EVs released from cells into the extracellular space [72]. Exosomes derived from the inward budding of the membrane in endosomes, forming intraluminal vesicles into multivesicular bodies (MVBs) that fuse with the plasma membrane and release exosomes into the extracellular space [73]. Exosome cargos, in response to different physiological or pathological conditions, can be formed by DNA, including ssDNA, dsDNA, genomic DNA, mtDNA, and even reverse-transcribed complementary DNAs [74]. To date, it seems clear how exosome heterogeneity depends on their biological cargo, function impacts on recipient cells, and cellular sources. That may have repercussions on their extraction; for example, isolated exosomes may contain subpopulations with a distinct size range. Therefore, an optimal method for the exosome isolations would require large pools of purification procedures, a high recovery yield, high purity of exosomes, and high efficiency [75]. Several exosomes isolation techniques have been developed, each exploiting a particular property of exosomes, such as their density, shape, size, and unique surface proteins, to aid their isolation [76]. Gene targeting can be performed through a plasmid with regulatory sequences, enabling the regulatory control of expression (inducible promoter) or as a PCR product [77]. A combination of all these aspects would have the potential to optimize the size-based separation techniques.

### 3.2. Vexosomes: A Novel Gene Delivery System

**Figure 1:**
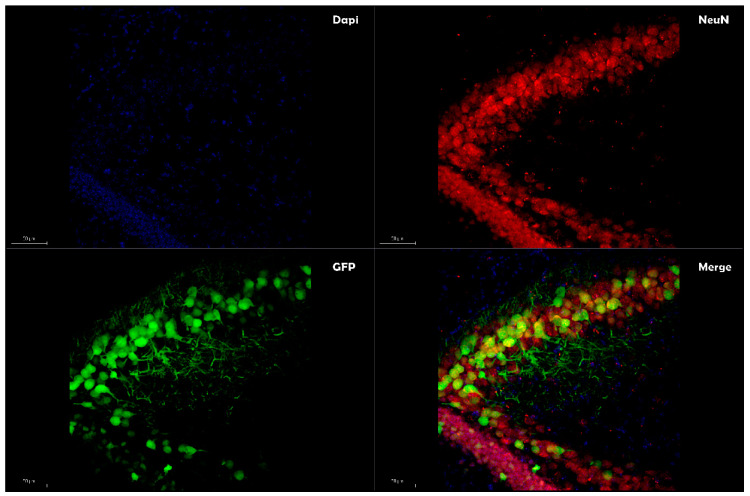
Exosome-enveloped adeno-associated viruses (AAVs) enhance transgene expressions in neurons. The co-localization of GFP (green) with the neuronal marker NeuN (merge) expressed by exosome-enveloped AAV vectors. Images were taken using a Leica SP8 confocal microscope at 20× magnification. Scale bar represents 50 μm.

**Figure 2:**
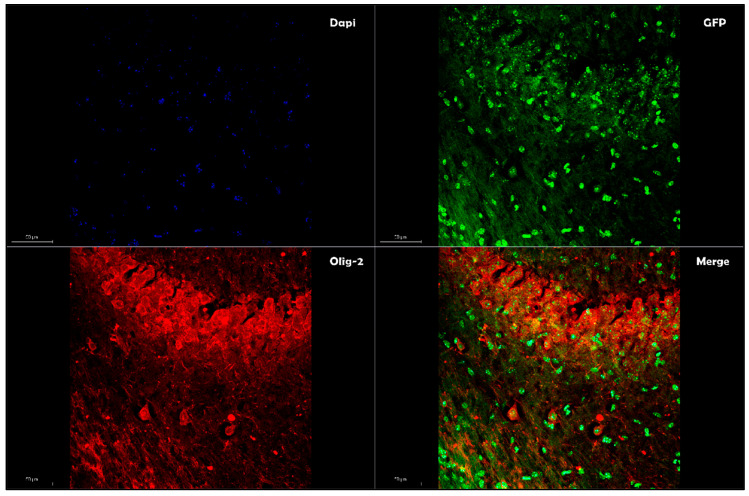
Exosome-enveloped AAVs enhance transgene expressions in oligodendrocytes. The colocalization of GFP (green) with oligodendrocytes marker Olig-2 (Merge) expressed by exosome-enveloped AAV vectors. Images were taken using a Leica SP8 confocal microscope at 20× magnification. Scale bar represents 50 μm.

Due to these functions, exosomes have brought to challenge the traditional classification of enveloped and nonenveloped viruses [78], providing enhanced transfection efficiencies in gene therapy. By combining the desirable features of both exosomes and the AAV vector system, ongoing research is aimed at understanding the role of the exosomes-enveloped viral vector (exo-AAV or vexosomes), where viral capsid has been coated with a surrounding, host-derived membrane, allowing enveloping of the protein viral vector to coordinate cellular tropism by binding to cell-surface molecules [79]. Exo-AAV can be achieved by transfecting the AAV vectors into human embryonic kidney cell line 293 (HEK-293T), and conditioned media containing the EVs associated with AAV vectors are isolated by density gradient centrifugation using iodixanol [80]. The initial characterization of this viral envelope by transmission electron microscopy exhibited an AAV/EVs association with the size range of ~50 to 200 nm [80]. Exo-AAV can also be engineered to display targeting peptides on their surfaces to enable enhanced deliveries to the target tissues. When injected systemically in mice, exo-AAV crossed the BBB and enabled efficient transduction in the central nervous system (CNS) [81]. Other studies based on exo-AAVs have reported an improvement of transduction profiles in different AAV serotype in vitro and in vivo conditions [72,82]. While György et al. [83] document that exo-AAV1 (the number denotes the capsid serotype) is a potent carrier of transgenes into cochlear and vestibular hair cells both in vitro and in vivo, Orefice et al. [84] reported an enhancement of the transgene expression resulting from two different AAV serotypes (AAV6 and AAV9) enveloped with exosomes restricted mainly to neurons (Figure 1) and oligodendrocytes (Figure 2). Clinical studies have reported that the trigger of neurodegenerative diseases can arise in a focus of genetically altered cells [72] and spread from one region of the CNS to another. In this context, the study by Orefice et al. revealed an interesting feature that might be imported to better optimize the exo-AVV like a valid therapeutic gene delivery. The continual expansion of miniaturized optical fiber-based endoscopes enabled real-time imaging to track the exo-AAV spread into the brain, showing it more widespread in the contralateral hemisphere than standard AAVs after intracerebral injections [84]. This study, added with previous studies conducted in the last few years, highlights the potential of using exo-AAVs for gene delivery, particularly to address the issue of diffusion limitations associated with large fragments of DNA to reach CNS cells far from the injection site. The strategy to envelope AAV vectors with exosome is also employed to circumvent a preexisting immunity to AAVs [85,86,87]; a study performed on female Balb/c mice exo-AAV9 has been proven effective to evade human Nabs against AAVs following the systemic administration [88]. Enveloped viral vectors with exosomes also offer significant advantages, both in reducing the number of injections required to achieve spreading into a targeted brain region (minimizing the risk of high-dose AAV administration-mediated toxicity) and delivering the dose rate needed to achieve the target concentration, possibly decreasing the vector doses required for therapeutic efficacy. All these characteristics make this subset of EVs a promising in vivo delivery system. Small RNAs (siRNA and miRNA) have been successfully loaded into EVs for different delivery applications [89,90,91,92]; however, the potential utility of different vesicles based on their biogenesis, size, content, and release pathway to load and deliver foreign DNA remains relatively unexplored.

## 4. Nucleic Acid-Loaded Extracellular Vesicles: Current Methods

In comparison to virus-derived vectors, exosomes have several advantages: mainly their cost-effectiveness, availability, and reduced risk of immune responses but, also, almost unlimited transgene sizes and the possibility of repeated administrations. However, due to the small sizes of exosomes, the capability to encapsulate plasmids with varied sizes is still a matter of debate of the ongoing research; thus, several strategies have been reframed to address this issue, as listed in Table 1. Finally, there is one more consideration to make concerning the difference between linear DNA and circular DNA (plasmid DNA). While linear DNA represents linear chromosomes that possess internal repeats of their terminal sequences forming intramolecular crossed-strand exchanges, allowing the replication of the chromosome ends [93], circular DNA is an independent, circular DNA vector that codes for gene expressions [94,95]. These differences affect choosing the transfection strategy to adopt for the experiment’s aim. DNA loading through transient transfection strategies results in more efficient with plasmid DNA compared to linear DNA [96], probably because circular DNA is not vulnerable to exonucleases, while linear DNA fragments are quickly degraded by these enzymes [95]. Contrary, stable transfections are more efficient when using linear DNA due to its optimal integration into the host genome [96].

### 4.1. Transfection-Mediated DNA Loading

Transfection is the procedure of a non-viral-mediated delivery of foreign genetic material into host cells [97]. Depending on the aim of the experimental research, it is essential to distinguish between transient and stable transfections. Through transient transfections, the transfected cells express the foreign gene, not integrating it into their genome. Therefore, the new gene will not perform DNA replication. These cells express the transiently transfected gene for a limited period—usually several days—after which, the foreign gene is lost through cell division or other factors [97]. Stably transfected cells begin with transient transfection, followed by an infrequent but essential process of serendipity. In a small proportion of transfected cells, the foreign gene has integrated into the genome to become part and, afterward, to be duplicated. The transfection-based technique has been conducted with exosomes derived from different cellular sources. The successful loading of exogenous genetic material in exosomes derived from murine dendritic cells has been performed by Seow et al. [98], while exosomes derived from HEK-293 cells were loaded with a plasmid containing GE11 peptide DNA [99]. As mentioned above, DNA loading through transfection strategies results in it being more efficient with plasmid DNA compared to linear DNA. However, some drawbacks have been raised in this technique; in particular, the issue has shifted to the remainders of the transfection reagents (like calcium phosphate and diethyl aminoethyl (DEAE)-dextran) that might cause incorrect or inefficient incorporations, leading to an undesirable downregulation of gene expressions in recipient cells. Similar importance is the localization of the transfection reagents [100]. The transfection reagents might remain partly hooked to the transfected acid nuclei to be (co)secreted in the culture medium. This may significantly affect the uptake behavior of the released transfected acid nuclei and its localization in either the exosome or protein complexes. Thoughtfulness should also be given to the complexity that characterizes the transfection process, from the initial DNA attachment to the plasma membrane and internalization via endocytosis, its release from the endosome followed by the dissociation of the vector from the DNA until its transfer into the nucleus, suggesting that an interplay of several essential parameters needs to be considered to achieve an efficient DNA delivery. Furthermore, due to their coprecipitation with exosomes at high centrifugal forces, complexes of transfection reagents and plasmid DNA might be mistakenly deducted as exosome-encapsulated acid nuclei. Following transfection, the risk of confounding the outcomes by analyzing the presence of foreign nucleic acid into exosomes is high; therefore, protocols that rigorously characterize the purification methods are still necessary.

### 4.2. Electroporation Procedure-Mediated DNA Loading into Extracellular Vesicles

The electroporation approach seems to guarantee a more reliable encapsulation of foreign genetic material, maintaining EVs integrity and functionality, and seems to be a viable alternative for cell types that are not responsive to the transfection method. An electrical pulse at an optimized voltage (1° C/pulse) has been applied to not damage the EVs membrane and overcome the cell membrane capacitance, disturbing the phospholipid bilayer of the membrane and creating transient membrane pores through which charged molecules like DNA cross [101]. Lamichhane and colleagues [102] reported exogenous linear foreign genetic material loaded into EVs using this technique in quantities sufficient for plasmid DNA. Besides, the authors established that the loading efficiency and capacity of linear DNA in EVs are dependent on the DNA size. Indeed, an increase of linear dsDNA fragments from 250 to 4000 bp in lengths was packaged into EVs through electroporation. Plasmid DNA fell within the range 750–1000 bp exhibiting low incorporation into EVs. One of the main advantages of electroporation is that it has a minimal effect on exosomal components such as ligands and receptors present on its membrane surface. However, as has been reported by Hood et al. [103] and Johnsen et al. [104] electroporation may trigger the aggregation of EVs and change their morphological characteristics. Thus, careful deductions need to be given while interpreting the loading using the electroporation method. The electroporation procedure can also induce cell toxicity; this issue might be minimized through experimental optimization methods and may be counterbalanced by increased transfection efficiencies. Another limitation of the electroporation technique is linked to the types of equipment that can vary among laboratories influencing the electroporation outcome and the electrical proprieties of the plasma membrane. Finally, cells are well-known to have a plasma membrane that consists of a phospholipid bilayer, which forms a stable barrier between two aqueous compartments and is a good electrical insulator [105]. This electrical property characterization of cells raises a question: According to the principle of electroporation that applies to all cells, how can its efficiency depend on the electrical circuit of the plasma membrane? The difference of conductivity in a cell represents an important matter to reflect during an electroporation approach [106].

### 4.3. Additional Methods Used for Loading in EVs: The Sonication and Saponin Methods

There is another method for DNA loading in EVs, such as sonication [107,108]. Sonication is the “cleanest” method, with a high loading efficiency, because it does not use enzymes or chemicals that might be carried throughout the sequencing workflow, negatively affecting the read quality. However, this method is restricted to the loading of smaller DNA molecules. Besides, the loading of DNA in EVs induces a prolonged release of catalase, as measured by retained the catalase activity over time [109]. Noteworthy, EVs loaded using the sonication approach do not show sound therapeutic effects in vivo, likely due to disruption of the exosomes’ integrity, making them more vulnerable to degradation via the reticuloendothelial system. Besides, after the loading process, the EVs integrity and the loss of intrinsic contents and biological properties deserve further attention. Although it has never been used to load plasmid DNA into EVs, another loading method consists of making permeable the EVs membranes through saponin use. Saponin is a detergent-like molecule able to interact with and remove cholesterol from EVs membranes, forming pores without leading to morphological and functional alterations of vesicles [110]. Indeed, even though the sonication and permeabilization method share similar loading efficiencies and sustained releases of EVs-encapsulated cargos, as mentioned above, EVs loaded by sonication appear to have undergone size and morphological alterations. The saponin permeabilization was utilized in a study assessing the use of protein catalase-loaded exosomes derived from macrophages as a drug delivery system for PD treatment [111]. An important aspect that emerged from this study, EVs loaded using saponin showed a prolonged release of their encapsulated cargos. However, although saponin permeabilization represents a straightforward loading method, it has never been wholly deepened in other preclinical and clinical studies. Moreover, an important saponin permeabilization-associated drawback consists of removing the residual trace of saponin after use, because, like many detergents, it can remain hooked to EVs, affecting their morphology.

## 5. DNA Loading Varies across Extracellular Vesicles Subsets

The discrepancies emerged concerning the content of different part-genomic DNA (gDNA) [112] and carrying and deliver nucleic acids to recipient cells depending on the EV subsets urge studies aimed at understanding whether the plasmid DNA loading could vary in different subsets of EVs. Kanada et al. [113] isolated exosomes and ectosomes from the same cell source (HEK293T), reporting a differential loading of nucleic acids between exosomes and MVs. Furthermore, they observed that ectosomes loaded with DNA were more efficient than exosomes delivering functional nucleic acids to target cells. These results are consistent with those reported by Lamichhane et al. [102], where, by electroporation-mediated DNA loading, they observed MVs exhibit an expanded capacity for effective linear DNA sizes (4–6 kb) than exosomes. This review agreed with previous studies indicating that the different capacities in loading and delivering exogenous DNA between exosomes (50–150 nm) and MVs (100–500 nm) might stem from their biogenesis differences. Whereas exosome-like EVs originate from MVBs inside cells [114], MVs are plasma membrane-derived vesicles; therefore, it might be possible that both EV subsets have a different lipid composition. Ectosomes and exosomes are endowed of the same membrane topology of donor cells [115], and consolidated evidence indicates that the composition of the lipid bilayer in exosomes differs from the lipid composition of MVs [116]. Besides, changes in the membrane lipid composition may alter the membrane fluidity and their curvature. This variability of the lipid compositions into exosomes and MVs might affect their permeability to electroporation-mediated DNA loading.

## 6. The Paradox of Persistence for DNA Plasmids and Viral Vectors

An enigma links plasmid DNA and AAV vectors, respectively: DNA-associated persistence and the persistence of the AAV genome. Plasmids are ubiquitous in nature; whereas plasmid DNA can be transmitted and replicated in the bacterial cell (horizontal gene transfer), chromosomal DNA can only vertically be transmitted between daughter and mother nuclei [117]. However, how plasmid DNA can persist in cell populations remains a crucial dilemma. Several studies have reported the unfavorable and favorable factors determining plasmid DNA persistence [118,119], proposing several hypotheses to explain plasmid persistence, including cross-ecotype transfer, host-plasmid coadaptation, plasmid hitchhiking, and high plasmid transfer rates. However, it is still missing a clear answer that adequately elucidates the plasmid paradox. Harrison et al. identified in chromids the source that may elucidate the paradox of plasmid-associated persistence [120]. Chromids are very large plasmids (generally > 500 kb) that frequently encode essential genes for core physiology [120], and there are about 10% of the bacterial genomes. Chromids are endowed of replication origins related to one of the plasmids, and thus, these elements retain many of the chromosomal and plasmid-like functional characteristics, like carrying essential housekeeping genes and sharing the codon usage properties of a chromosome [121]. This chromid configuration may allow the host cell to keep a larger genome while allowing the chromosome to remain smaller. In this condition, genetic material can be quickly replicated [122]. How are long-term AAV transgene expressions? The expressions of genes delivered by AAV can persist long-term [123]; due to the absence of rep gene products, the vector genome does not undergo site-specific integration in the host DNA. rAAV genomes have been documented to persist for six and three-point seven years in preclinical-clinical trials, respectively [124]. Recently it has been proposed that the rAAV genome’s ability to persist into host cells relies on cellular proteins that convert the single- to double-stranded DNA. The resultant duplex DNA genome, through either intra or intermolecular recombination at the ITRs, leads to circular episomes or linear concatamers [125]. These resultant episomal forms of rAAVs are thought to be the factors responsible for the long-term gene expression in the nucleus of transfected cells. The paradox of the persistence of plasmid DNA and rAAV still needs to be substantiated with more studies; thus, this review suggests a possible investigation that is to go beyond the study of plasmids as individual entities and analyze them in a more integrated view, considering whether and how interactions interfere with viral genetic elements.

## 7. Exogenous DNA Loading Elicits a Different Level of Intrinsic Cellular Response

An additional important point regarding exogenous DNA loading has not been thoroughly addressed in this review: the different cellular responses induced by the transfected DNA. As mentioned above, the introduction of plasmid DNA into mammalian cells is not fallowed by its complete integration inside the genome, but it persists as extrachromosomal DNA for a period. Despite that introduced plasmid DNA is subjected to degradation, some of it can acquire a nucleosome structure; a study conducted by Igoucheva et al. [126] reported cellular responses to transfected dsDNA, which vary according to cell types. Moreover, following the introduction of dsDNA into mammalian cells, the authors observed the transcription of several genes responsible for sensing DNA damage and repair. This led to the suggestion that mammalian cells might trigger a response to the presence of dsDNA. More elucidation of cellular responses will help interpret the episomal gene-targeting process better and give answers to another important question: What prevents the complete integration of plasmid DNA inside the genome? Productive transfection and gene delivery request that plasmid DNA can enter into transfected cells, and subsequently, several intracellular processes allow the DNA to move from the extracellular surface to inside of the cell and through the cytoplasm to reach the nucleus before any transcription can initiate. However, it has been estimated that only a small amount of plasmid DNA reaches the nucleus in transfected cells following lipofection [127]. The cytoplasm’s role is considered as a possible cause for the incomplete integration of DNA into the nucleus. The cytoplasm is a viscous gel-like substance enclosed within the cell membrane that results in decreased mobility of the macromolecules [127]. Several studies have demonstrated that small solutes can diffuse freely and rapidly in the cytoplasm and the nucleus [128,129]. Additionally, studies performed to assess plasmid DNA fragment movements have reported that, while small DNA can diffuse, those larger than 2000 bp were effectively unable to diffuse to any degree in the cytoplasm in any reasonable physiological time frame [128]. The existence of the cytoskeleton and the large numbers of actin filaments, microtubules, and intermediate filaments that form a highly cross-linked gel-sol might be responsible for the diffusion limitation [130]. Due to this system of networks, if DNA is released at a site distant from the nucleus, it cannot move toward its desired location. This has been demonstrated in the case of liposome transfections, where some DNA is left free in the cytoplasm and never reaches the nucleus [131]. This brings to consider whether cytoplasmic trafficking is sufficient since there does not permit the diffusion and entrance of large DNA, even though strategies to load large DNA fragments up to 167 kb in length will be discovered.

## 8. Emerging DNA-Loading Strategies and Perspectives

Considerable interest is addressed in developing nonviral lipid-based vectors due to their potential to load and transfer a large amount of DNA into cells. Cationic-lipid (CL)-based vectors are synthetic carries of nucleic acids currently employed in gene delivery, like CL-DNA [132], and gene silencing, like the CL-siRNA complex [133]. No immunogenicity, low toxicity, and ease of production are the characteristics that distinguish these nonviral vectors. However, their transfection and silencing efficiencies remain low compared to those of viral vectors. EVs-associated artificial nanoparticle systems are aimed at mimicking their properties and may represent a valid strategy to overcome the EVs-associated limitations. Several types of nano-based drug delivery systems (DDSs) are currently under consideration for drug-targeting applications. Liposomes have been the first to obtain clinical approval [134]; they are the most biocompatible and least toxic artificial systems, constructed by the main components of cell membranes: phospholipids and cholesterol. In addition to their non-toxic and biocompatible natures, they can load drugs and prevent biodegradation. Further details of liposomes will be discussed in Section “Biomimetic Vesicles Frontier”.

Exosomes have many similarities with liposomes [135]; this has allowed researchers to improve the methodologies aimed at increasing the targeting potential of ligand-targeted liposomes. In this context, through the procedures applied in the liposome technology field, hybrid exosomes engineered by fusions with liposomes represent a current interesting research frontier [135,136]. The strategy to embed exosomes with a specific membrane protein isolated from genetically modified cells and fuse them with various liposomes is offering the advantage to enhance the encapsulation efficiency of plasmid DNA, overcoming the size limitation observed with standard exosomes [136,137].

### Biomimetic Vesicles Frontier

Inspired by biological cells’ architecture, considerable attention has been paid to assembling multicompartment systems using a biomimetic approach in the last decade. The field of biomimetics has opened a new frontier of a wide range of biomedical applications aimed at improving patient compliance with low system side effects. Considerable progress has been achieved in reconstructing and assembling multicompartment systems based on liposomes composed of self-assembled bilayers of phospholipids, the active compound of the architecture of cell membranes that makes them advantageous in terms of a lack of toxicity, biodegradation, and biocompatibility [138,139].

Due to their internal architecture of phospholipid bilayers, two different types of liposomes can be distinguished: unilamellar and multilamellar. Unilamellar liposomes consist of a single phospholipid bilayer that forms a physical barrier between the internal compartment (aqueous core) and the external environment, with particle sizes ranging from 0.1 to one micrometer. Instead, multilamellar liposomes are formed by several concentric phospholipid bilayers, with particle sizes until 100 micrometers [140]. Several approaches have been pursued to assemble these biomimetic vesicles, including sonication, reverse-phase evaporation, and membrane extrusion [141]. The first model of a liposome-based biomimetic, named vesosomes, derives from the fusion of unilamellar liposomes snared within a larger liposome, reported by Zasadzinski et al. [142]. Given the presence of hydrophobic and hydrophilic domains, these vesicles can load a variety of cargo, like DNA, enzymes, proteins, and drugs [143,144,145]. Moreover, due to the interior architecture that provides confined environments, the internal cargo will not be exposed to degradation under biologically relevant conditions. The multicompartment architecture of vesosomes offers the advantage to overcome the unilamellar liposome-associated drawbacks, such as the premature release of the cargo. The creation of multicompartmentalized vesicles has recently inspired researchers to perform the incorporation of hydrophobic and hydrophilic domains of liposomes into polymer films to form caposomes (>1 µm diameter) [146]. These biomimetic carriers assembled by most liposomal compartments afford the double advantage of enhancing the physical integrity of liposomes susceptible to enzyme degradation while also retaining their biomedical properties. The potential use of caposomes as a protein delivery carrier has been demonstrated by several studies [147,148]; special mention must be made to studies in which caposomes were employed to encapsulate the brain-derived neurotrophic factor (BDNF) for the treatment of neurological disorders [149]. So far, biomimetic vesicle-associated drawbacks still limit their complete commercialization in clinical applications. Stability represents the main issue for biomimetic vesicles because these carriers usually are assembled to be stored longer than one year. Several efforts have been performed to overcome this aspect; the replacement of phospholipids with sphingolipids has undoubtedly enhanced the stability over time; however, its prolonged exposure induces toxicity and inflammation response. Even though other modifications led to assembling liposomes like cubosomes or ufasomes, these formulations showed issues in other aspects, such as the loading and encapsulations of cargos.

## 9. Conclusions

Even though ongoing research is focused on optimizing the purification and analytical procedures for the study of EVs, the loading of plasmid DNA keeps being limited to a particular size of nucleic acids. Likely, a deeper understanding of the biological origin of DNA inside EVs might be relevant in enhancing further methodologies of loading, making EVs-efficient foreign DNA delivery systems.

## Figures and Tables

**Table 1:** Comparison of the exogenous methods for plasmid DNA loading into extracellular vesicles (EVs).

Method	Advantages	Disadvantages	References
Transfection	○ High efficiency ○ Easy to apply ○ Applied in most of the eukaryotic cells ○ A high concentration of plasmid DNA	○ Transfection reagents partly hooked ○ Potential deformation of vesicle membranes ○ Low efficiency in suspension cells	[97,98,99,100]
Electroporation	○ Loading large plasmid DNA ○ Applied to cells resistant to transfection method ○ Plasmid DNA is propelled directly into the target cells ○ Versatility	○ Equipment varies among laboratories ○ Cells damage ○ Nonspecific transport	[101,103,104,105]
Sonication	○ Increased loading efficiency (compared to other methods) ○ Not need enzymes or chemicals reagents	○ Potential deformation of the membrane through overheating ○ Expensive equipment	[107,108,109]
Saponin permeabilization	○ Reversible ○ Not permeabilize the nuclear membrane ○ Used to selectively permeabilize mammalian cell membranes based on their cholesterol content and concentration of saponin	○ Performed in a handful of studies ○ Membrane damage	[110,111]

## References

[1] Deverman B.E., Ravina B.M., Bankiewicz K.S., Paul S.M., Sah D.W.Y. (2018). Gene therapy for neurological disorders: Progress and prospects. Nat. Rev. Drug Discov..

[2] Ventola C.L. (2017). Progress in nanomedicine: Approved and investigational nanodrugs. Pharm. Ther..

[3] Du Rietz H., Hedlund H., Wilhelmson S., Nordenfelt P., Wittrup A. (2020). Imaging small molecule-induced endosomal escape of siRNA. Nat. Commun..

[4] Daya S., Berns K.I. (2008). Gene therapy using adeno-associated virus vectors. Clin. Microbiol. Rev..

[5] Lai C.M., Lai Y.K., Rakoczy P.E. (2002). Adenovirus and adeno-associated virus vectors. DNA Cell Biol..

[6] Wold W.S., Toth K. (2013). Adenovirus vectors for gene therapy, vaccination and cancer gene therapy. Curr. Gene Ther..

[7] Okada Y., Okada N., Mizuguchi H., Hayakawa T., Mayumi T., Mizuno N. (2003). An investigation of adverse effects caused by the injection of high-dose TNFα-expressing adenovirus vector into established murine melanoma. Gene Ther..

[8] Gregory S.M., Nazir S.A., Metcalf J.P. (2011). Implications of the innate immune response to adenovirus and adenoviral vectors. Future Virol..

[9] Yang Y., Li Q., Ertl H.C., Wilson J.M. (1995). Cellular and humoral immune responses to viral antigens create barriers to lung-directed gene therapy with recombinant adenoviruses. J. Virol..

[10] Atasheva S., Shayakhmetov D.M. (2016). Adenovirus sensing by the immune system. Curr. Opin. Virol..

[11] Worgall S., Wolff G., Falck-Pedersen E., Crystal R.G. (1997). Innate immune mechanisms dominate elimination of adenoviral vectors following in vivo administration. Hum. Gene Ther..

[12] Marshall E. (2000). Gene therapy on trial. Science.

[13] Muruve A.D., Barnes M.J., Stillman I.E., Libermann T.A. (1999). Adenoviral gene therapy leads to rapid induction of multiple chemokines and acute neutrophil-dependent hepatic injury in vivo. Hum. Gene Ther..

[14] Kuzmin I.A., Finegold M.J., Eisensmith R.C. (1997). Macrophage depletion increases the safety, efficacy and persistence of adenovirus-mediated gene transfer in vivo. Gene Ther..

[15] Lieber A., He C.Y., Meuse L., Schowalter D., Kirillova I., Winther B., Kay M.A. (1997). The role of Kupffer cell activation and viral gene expression in early liver toxicity after infusion of recombinant adenovirus vectors. J. Virol..

[16] Lozier J.N., Metzger M.E., Donahue R.E., Morgan R.A. (1999). Adenovirus-Mediated expression of human coagulation factor IX in the rhesus macaque is associated with dose-limiting toxicity. Blood.

[17] Shayakhmetov D.M., Li Z.Y., Ni S., Lieber A. (2004). Analysis of adenovirus sequestration in the liver, transduction of hepatic cells, and innate toxicity after injection of fiber-modified vectors. J. Virol..

[18] Tao N., Gao G.P., Parr M., Johnston J., Baradet T., Wilson J.M., Barsoum J., Fawell S.E. (2001). Sequestration of adenoviral vector by Kupffer cells leads to a nonlinear dose response of transduction in liver. Mol. Ther..

[19] Wright J.F. (2008). Manufacturing and characterizing AAV-based vectors for use in clinical studies. Gene Ther..

[20] Drouin L.M., Agbandje-McKenna M. (2013). Adeno-Associated virus structural biology as a tool in vector development. Future Virol..

[21] Büning H., Srivastava A. (2019). Capsid modifications for targeting and improving the efficacy of AAV Vectors. Mol. Ther. Methods Clin. Dev..

[22] Aponte-Ubillus J., Barajas D., Peltier J., Bardliving C., Shamlou P., Gold D. (2018). Molecular design for recombinant adeno-associated virus (rAAV) vector production. Appl. Microbiol. Biotechnol..

[23] Penaud-Budloo M., François A., Clément N., Ayuso E. (2018). Pharmacology of recombinant adeno-associated virus production. Mol. Ther. Methods Clin. Dev..

[24] (2018). FDA approves hereditary blindness gene therapy. Nat. Biotechnol..

[25] Strimvelis K., Yescarta L. (2019). Gene therapy’s next installment. Nat. Biotechnol..

[26] Bainbridge J.W.B., Mehat M.S., Sundaram V., Robbie S.J., Barker S.E., Ripamonti C., Georgiadis A., Mowat F.M., Beattie S.G., Gardner P.J. (2015). Long-Term effect of gene therapy on Leber’s congenital amaurosis. N. Engl. J. Med..

[27] Russell S., Bennett J., A Wellman J., Chung D.C., Yu Z.-F., Tillman A., Wittes J., Pappas J., Elci O., McCague S. (2017). Efficacy and safety of voretigene neparvovec (AAV2-hRPE65v2) in patients with RPE65-mediated inherited retinal dystrophy: A randomised, controlled, open-label, phase 3 trial. Lancet.

[28] Solinís M.Á., del Pozo-Rodríguez A., Apaolaza P.S., Rodríguez-Gascón A. (2015). Treatment of ocular disorders by gene therapy. Eur. J. Pharm. Biopharm..

[29] Ertl H.C.J., High K.A. (2017). Impact of AAV capsid-specific T-Cell responses on design and outcome of clinical gene transfer trials with recombinant adeno-associated viral vectors: An evolving controversy. Hum. Gene Ther..

[30] Vandamme C., Adjali O., Mingozzi F. (2017). Unraveling the complex story of immune responses to AAV vectors trial after trial. Hum. Gene Ther..

[31] Clement N., Grieger J.C. (2016). Manufacturing of recombinant adeno-associated viral vectors for clinical trials. Mol. Ther. Methods Clin. Dev..

[32] Shin J.H., Yue Y., Smith B., Duan D. (2012). Humoral immunity to AAV-6, 8, and 9 in normal and dystrophic dogs. Hum. Gene Ther..

[33] Arnett A.L., Garikipati D., Wang Z., Tapscott S., Chamberlain J.S. (2011). Immune responses to rAAV6: The influence of canine parvovirus vaccination and neonatal administration of viral vector. Front. Microbiol..

[34] Rapti K., Louis-Jeune V., Kohlbrenner E., Ishikawa K., Ladage D., Zolotukhin S., Hajjar R.J., Weber T. (2012). Neutralizing antibodies against AAV serotypes 1, 2, 6, and 9 in sera of commonly used animal models. Mol. Ther..

[35] Sun L., Li J., Xiao X. (2000). Overcoming adeno-associated virus vector size limitation through viral DNA heterodimerization. Nat. Med..

[36] Dong J.Y., Fan P.D., Frizzell R.A. (1996). Quantitative analysis of the packaging capacity of recombinant adeno-associated virus. Hum. Gene Ther..

[37] Tse L.V., Moller-Tank S., Asokan A. (2015). Strategies to circumvent humoral immunity to adeno-associated viral vectors. Expert Opin. Biol. Ther..

[38] Li C., Samulski R.J. (2020). Engineering adeno-associated virus vectors for gene therapy. Nat. Rev. Genet..

[39] György B., Maguire C.A. (2018). Extracellular vesicles: Nature’s nanoparticles for improving gene transfer with adeno-associated virus vectors. Wiley Interdiscip. Rev. Nanomed. Nanobiotechnol..

[40] Théry C., Witwer K.W., Aikawa E., Alcaraz M.J., Anderson J.D., Andriantsitohaina R., Antoniou A., Arab T., Archer F., Atkin-Smith G.K. (2018). Minimal information for studies of extracellular vesicles 2018 (MISEV2018): A position statement of the International Society for Extracellular Vesicles and update of the MISEV2014 guidelines. J. Extracell. Vesicles.

[41] Noble J.M., Roberts L.M., Vidavsky N., Chiou A.E., Fischbach C., Paszek M.J., Estroff L.A., Kourkoutis L.F. (2020). Direct comparison of optical and electron microscopy methods for structural characterization of extracellular vesicles. J. Struct. Biol..

[42] Cufaro M.C., Pieragostino D., Lanuti P., Rossi C., Cicalini I., Federici L., De Laurenzi V., Del Boccio P. (2019). Extracellular vesicles and their potential use in monitoring cancer progression and therapy: The contribution of proteomics. J. Oncol..

[43] Kalluri R., LeBleu V.S. (2020). The biology, function, and biomedical applications of exosomes. Science.

[44] Mathieu M., Martin-Jaular L., Lavieu G., Théry C. (2019). Specificities of secretion and uptake of exosomes and other extracellular vesicles for cell-to-cell communication. Nat. Cell Biol..

[45] Bebelman M.P., Smit M.J., Pegtel D.M., Baglio S.R. (2018). Biogenesis and function of extracellular vesicles in cancer. Pharmacol. Ther..

[46] Doyle L.M., Wang M.Z. (2019). Overview of extracellular vesicles, their origin, composition, purpose, and methods for exosome isolation and analysis. Cells.

[47] Caruso S., Poon I.K.H. (2018). Apoptotic cell-derived extracellular vesicles: More than just debris. Front. Immunol..

[48] Guescini M., Genedani S., Stocchi V., Agnati L.F. (2010). Astrocytes and Glioblastoma cells release exosomes carrying mtDNA. J. Neural. Transm..

[49] Bowles D.E., McPhee S.W., Li C., Gray S.J., Samulski J.J., Camp A.S., Li J., Wang B., Monahan P.E., Rabinowitz J.E. (2012). Phase 1 gene therapy for Duchenne muscular dystrophy using a translational optimized AAV vector. Mol. Ther..

[50] Thakur B.K., Zhang H., Becker A., Matei I., Huang Y., Costa-Silva B., Zheng Y., Hoshino A., Brazier H., Xiang J. (2014). Double-Stranded DNA in exosomes: A novel biomarker in cancer detection. Cell Res..

[51] Ramirez S.H., Andrews A.M., Paul D., Pachter J.S. (2018). Extracellular vesicles: Mediators and biomarkers of pathology along CNS barriers. Fluids Barriers Cns.

[52] O’Brien K.P., Breyne K., Ughetto S., Laurent L.C., Breakefield X.O. (2020). RNA delivery by extracellular vesicles in mammalian cells and its applications. Nat. Rev. Mol. Cell Biol..

[53] Schultz B.R., Chamberlain J.S. (2008). Recombinant adeno-associated virus transduction and integration. Mol. Ther..

[54] Gernoux G., Wilson J.M., Mueller C. (2017). Regulatory and exhausted T Cell responses to AAV capsid. Hum. Gene Ther..

[55] Guo P., Zhang J., Chrzanowski M., Huang J., Chew H., Firrman J.A., Sang N., Diao Y., Xiao W. (2018). Rapid AAV-Neutralizing antibody determination with a Cell-Binding assay. Mol. Ther. Methods Clin. Dev..

[56] Wang D., Zhong L., Li M., Li J., Tran K., Ren L., He R., Xie J., Moser R.P., Fraser C. (2018). Adeno associated virus neutralizing antibodies in large animals and their impact on brain intraparenchymal gene transfer. Mol. Ther. Methods Clin. Dev..

[57] Karman J., Gumlaw N.K., Zhang J., Jiang J.L., Cheng S.H., Zhu Y. (2012). Proteasome inhibition is partially effective in attenuating pre-existing immunity against recombinant adeno-associated viral vectors. PLoS ONE.

[58] Mimuro J., Mizukami H., Hishikawa S., Ikemoto T., Ishiwata A., Sakata A., Ohmori T., Madoiwa S., Ono F., Ozawa K. (2013). Minimizing the inhibitory effect of neutralizing antibody for efficient gene expression in the liver with adeno-associated virus 8 vectors. Mol. Ther..

[59] Ayuso E., Mingozzi F., Montane J., Leon X., Anguela X.M., Haurigot V., Edmonson S.A., Africa L., Zhou S., High K.A. (2010). High AAV vector purity results in serotype and tissue-independent enhancement of transduction efficiency. Gene Ther..

[60] Chamberlain K., Riyad J.M., Weber T. (2016). Expressing transgenes that exceed the packaging capacity of adeno-associated virus capsids. Hum. Gene Ther. Methods.

[61] Pryadkina M., Lostal W., Bourg N., Charton K., Roudaut C., Hirsch M.L., Richard I. (2015). A comparison of AAV strategies distinguishes overlapping vectors for efficient systemic delivery of the 6.2 kb Dysferlin coding sequence. Mol. Ther. Methods Clin. Dev..

[62] Ding W., Zhang L., Yan Z., Engelhardt J.F. (2005). Intracellular trafficking of adeno-associated viral vectors. Gene Ther..

[63] Trapani I. (2019). Adeno-Associated viral vectors as a tool for large gene delivery to the retina. Genes.

[64] Carvalho L.S., Turunen H.T., Wassmer S.J., Luna-Velez M.V., Xiao R., Bennett J., Vandenberghe L.H. (2017). Evaluating efficiencies of dual AAV approaches for retinal targeting. Front. Neurosci..

[65] Sharma P., Mesci P., Carromeu C., McClatchy D.R., Schiapparelli L., Yates J.R., Muotri A.R., Cline H.T. (2019). Exosomes regulate neurogenesis and circuit assembly. Proc. Natl. Acad. Sci. USA.

[66] Vlassov A.V., Magdaleno S., Setterquist R., Conrad R. (2012). Exosomes: Current knowledge of their composition, biological functions, and diagnostic and therapeutic potentials. Biochim. Biophys. Acta.

[67] Lim Y.-J., Lee S.-J. (2017). Are exosomes the vehicle for protein aggregate propagation in neurodegenerative diseases?. Acta Neuropathol. Commun..

[68] Davis A.A., Leyns C.E.G., Holtzman D.M. (2018). Intercellular spread of protein aggregates in neurodegenerative disease. Annu. Rev. Cell Dev. Biol..

[69] Sweeney P., Park H., Baumann M., Dunlop J., Frydman J., Kopito R., McCampbell A., Leblanc G., Venkateswaran A., Nurmi A. (2017). Protein misfolding in neurodegenerative diseases: Implications and strategies. Transl. Neurodegener..

[70] Goto Y., Ogawa Y., Tsumoto H., Miura Y., Nakamura T.J., Ogawa K., Akimoto Y., Kawakami H., Endo T., Yanoshita R. (2018). Contribution of the exosome-associated form of secreted endoplasmic reticulum aminopeptidase 1 to exosome-mediated macrophage activation. Biochim. Biophys. Acta Mol. Cell Res..

[71] Wang M., Kaufman R. (2016). Protein misfolding in the endoplasmic reticulum as a conduit to human disease. Nature.

[72] Frank S.A. (2010). Evolution in health and medicine Sackler colloquium: Somatic evolutionary genomics: Mutations during development cause highly variable genetic mosaicism with risk of cancer and neurodegeneration. Proc. Natl. Acad. Sci. USA.

[73] Murphy D.E., De Jong O.G., Brouwer M., Wood M.J., Lavieu G., Schiffelers R., Vader P. (2018). Extracellular vesicle-based therapeutics: Natural versus engineered targeting and trafficking. Exp. Mol. Med..

[74] Zaborowski M.P., Balaj L., Breakefield X.O., Lai C.P. (2015). Extracellular vesicles: Composition, biological relevance, and methods of study. Bioscience.

[75] Tschuschke M., Kocherova I., Bryja A., Mozdziak P., Volponi A.A., Janowicz K., Sibiak R., Piotrowska-Kempisty H., Iżycki D., Bukowska D. (2020). Inclusion biogenesis, methods of isolation and clinical application of human cellular exosomes. J. Clin. Med..

[76] Li P., Kaslan M., Lee S.H., Yao J., Gao Z. (2017). Progress in exosome isolation techniques. Theranostics.

[77] Kallunki T., Barisic M., Jäättelä M., Liu B. (2019). How to choose the right inducible gene expression system for mammalian studies?. Cells.

[78] Firquet S., Beaujard S., Lobert P.-E., Sane F., Caloone D., Izard D., Hober D. (2015). Survival of enveloped and non-enveloped viruses on inanimate surfaces. Microbes Environ..

[79] Hudry E., Martin C., Gandhi S., György B., Scheffer D.I., Mu D., Merkel S.F., Mingozzi F., Fitzpatrick Z., Dimant H. (2016). Exosome-Associated AAV vector as a robust and convenient neuroscience tool. Gene Ther..

[80] Saari H., Turunen T., Lõhmus A., Turunen M., Jalasvuori M., Butcher S.J., Ylä-Herttuala S., Viitala T., Cerullo V., Siljander P. (2020). Extracellular vesicles provide a capsid-free vector for oncolytic adenoviral DNA delivery. J. Extracell. Vesicles.

[81] Wood M.J., O’Loughlin A.J., Samira L. (2011). Exosomes and the blood-brain barrier: Implications for neurological diseases. Ther. Deliv..

[82] Hudry E., Andres-Mateos E., Lerner E.P., Volak A., Cohen O., Hyman B.T., Maguire C.A., Vandenberghe L.H. (2018). Efficient gene transfer to the central nervous system by Single-Stranded Anc80L65. Mol. Ther. Methods Clin. Dev..

[83] György B., Sage C., Indzhykulian A., Scheffer D.I., Brisson A.R., Tan S., Wu X., Volak A., Mu D., Tamvakologos P.I. (2017). Rescue of hearing by gene delivery to inner-ear hair cells using exosome-associated AAV. Mol. Ther..

[84] Orefice N., Souchet B., Braudeau J., Alves S., Piguet F., Collaud F., Ronzitti G., Tada S., Hantraye P., Mingozzi F. (2019). Real-Time monitoring of exosome Enveloped-AAV Spreading by endomicroscopy approach: A new tool for gene delivery in the brain. Mol. Ther. Methods Clin. Dev..

[85] György B., Fitzpatrick Z., Crommentuijn M.H., Mu D., Maguire C.A. (2014). Naturally enveloped AAV vectors for shielding neutralizing antibodies and robust gene delivery in vivo. Biomaterials.

[86] Schiller L.T., Lemus-Diaz N., Rinaldi Ferreira R., Böker K.O., Gruber J. (2018). Enhanced production of Exosome-Associated AAV by overexpression of the Tetraspanin CD9. Mol. Ther. Methods Clin. Dev..

[87] Wassmer S.J., Carvalho L.S., György B., Vandenberghe L.H., Maguire C.A. (2017). Exosome-Associated AAV2 vector mediates robust gene delivery into the murine retina upon intravitreal injection. Sci. Rep..

[88] Meliani A., Boisgerault F., Fitzpatrick Z., Marmier S., Leborgne C., Collaud F., Sola M.S., Charles S., Ronzitti G., Vignaud A. (2017). Enhanced liver gene transfer and evasion of preexisting humoral immunity with exosome-enveloped AAV vectors. Blood Adv..

[89] Valadi H., Ekström K., Bossios A., Sjöstrand M., Lee J.J., Lötvall J. (2007). Exosome-Mediated transfer of mRNAs and microRNAs is a novel mechanism of genetic exchange between cells. Nat. Cell Biol..

[90] Alvarez-Erviti L., Seow Y., Yin H., Betts C., Lakhal S., Wood M.J.A. (2011). Delivery of siRNA to the mouse brain by systemic injection of targeted exosomes. Nat. Biotechnol..

[91] Zhang D., Lee H., Jin Y. (2020). Delivery of functional small RNAs via extracellular vesicles in vitro and in vivo. Methods Mol. Biol..

[92] Zhao L., Gu C., Gan Y., Shao L., Chen H., Zhu H. (2020). Exosome-Mediated siRNA delivery to suppress postoperative breast cancer metastasis. J. Control. Release.

[93] Eilebrecht S., Hotz-Wagenblatt A., Sarachaga V., Burk A., Falida K., Chakraborty D., Nikitina E., Tessmer C., Whitley C., Sauerland C. (2018). Expression and replication of virus-like circular DNA in human cells. Sci. Rep..

[94] Cohen R.N., van der Aa M.A., Macaraeg N., Lee A.P., Szoka F.C. (2009). Quantification of plasmid DNA copies in the nucleus after lipoplex and polyplex transfection. J. Control. Release.

[95] Peeva V., Blei D., Trombly G., Corsi S., Szukszto M.J., Rebelo-Guiomar P., Gammage P.A., Kudin A.P., Becker C., Altmüller J. (2018). Linear mitochondrial DNA is rapidly degraded by components of the replication machinery. Nat. Commun..

[96] Nafissi N., Alqawlaq S., Lee E.A., Foldvari M., Spagnuolo P.A., Slavcev R.A. (2014). DNA ministrings: Highly safe and effective gene delivery vectors. Mol. Ther. Nucleic Acids.

[97] Kim T.K., Eberwine J.H. (2010). Mammalian cell transfection: The present and the future. Anal. Bioanal. Chem..

[98] Seow Y., Alvarez-Erviti L., Matthew JWood M.J. (2009). Targeted delivery of plasmid DNA and siRNA with modified dendritic Cell-Derived Exosomes. Mol. Ther..

[99] Ohno S., Takanashi M., Sudo K., Ueda S., Ishikawa A., Matsuyama N. (2013). Systemically injected exosomes targeted to EGFR deliver antitumor microRNA to breast cancer cells. Mol. Ther..

[100] Gulick T. (2003). Transfection using DEAE-dextran. Curr. Protoc. Cell Biol..

[101] Sharifi Tabar M., Hesaraki M., Esfandiari F., Sahraneshin Samani F., Vakilian H., Baharvand H. (2015). Evaluating electroporation and lipofectamine approaches for transient and stable transgene expressions in human fibroblasts and embryonic stem cells. Cell J..

[102] Lamichhane T.N., Raiker R.S., Jay S.M. (2015). Exogenous DNA loading into extracellular vesicles via electroporation is size-dependent and enables limited gene delivery. Mol. Pharm..

[103] Hood J.L., Scott M.J., Wickline S.A. (2014). Maximizing exosome colloidal stability following electroporation. Anal. Biochem..

[104] Johnsen K.B., Gudbergsson J.M., Skov M.N., Christiansen G., Gurevich L., Moos T., Duroux M. (2016). Evaluation of electroporation-induced adverse effects on adipose-derived stem cell exosomes. Cytotechnology.

[105] Liang W., Zhao Y., Liu L., Wang Y., Li W.J., Lee G.B. (2017). Determination of cell membrane capacitance and conductance via optically induced electrokinetics. Biophys. J..

[106] Pavlin M., Miklavcic D. (2003). Effective conductivity of a suspension of permeabilized cells: A theoretical analysis. Biophys. J..

[107] Tang T.T., Lv L.L., Lan H.Y., Liu B.C. (2019). Extracellular vesicles: Opportunities and challenges for the treatment of renal diseases. Front. Physiol..

[108] Liao W., Du Y., Zhang C., Pan F., Yao Y., Zhang T., Peng Q. (2018). Exosomes: The next generation of endogenous nanomaterials for advanced drug delivery and therapy. Acta Biomater..

[109] Liu C., Su C. (2019). Design strategies and application progress of therapeutic exosomes. Theranostics.

[110] Sudji I.R., Subburaj Y., Frenkel N., García-Sáez A.J., Wink M. (2015). Membrane disintegration caused by the steroid saponin digitonin is related to the presence of cholesterol. Molecules.

[111] Haney M.J., Klyachko N.L., Zhao Y., Gupta R., Plotnikova E.G., He Z., Patel T., Piroyan A., Sokolsky M., Kabanov A.V. (2015). Exosomes as drug delivery vehicles for Parkinson’s disease therapy. J. Control. Release.

[112] Psifidi A., Dovas C.I., Bramis G., Lazou T., Russel C.L., Arsenos G., Banos G. (2015). Comparison of eleven methods for genomic DNA extraction suitable for large-scale whole-genome genotyping and long-term DNA banking using blood samples. PLoS ONE.

[113] Kanada M., Bachmann M.H., Hardy J.W., Frimannson D.O., Bronsart L., Wang A., Sylvester M.D., Schmidt T.L., Kaspar R.L., Butte M.J. (2015). Differential fates of biomolecules delivered to target cells via extracellular vesicles. Proc. Natl. Acad. Sci. USA.

[114] Antonyak M.A., Cerione R.A. (2015). Emerging picture of the distinct traits and functions of microvesicles and exosomes. Proc. Natl. Acad. Sci. USA.

[115] Choi D.S., Kim D.K., Kim Y.K., Gho Y.S. (2015). Proteomics of extracellular vesicles: Exosomes and ectosomes. Mass Spectrom. Rev..

[116] Haraszti R.A., Didiot M.-C., Sapp E., Leszyk J., Shaffer S.A., Rockwell H.E., Gao F., Narain N.R., DiFiglia M., Kiebish M.A. (2016). High-Resolution proteomic and lipidomic analysis of exosomes and microvesicles from different cell sources. J. Extracell. Vesicles.

[117] Lacroix B., Citovsky V. (2016). Transfer of DNA from bacteria to eukaryotes. mBio.

[118] Wein T., Hülter N.F., Mizrahi I., Dagan T. (2019). Emergence of plasmid stability under non-selective conditions maintains antibiotic resistance. Nat. Commun..

[119] De Gelder L., Williams J.J., Ponciano J.M., Sota M., Top E.M. (2008). Adaptive plasmid evolution results in host-range expansion of a broad-host-range plasmid. Genetics.

[120] Harrison P.W., Lower R.P., Kim N.K., Young J.P. (2010). Introducing the bacterial ‘chromid’: Not a chromosome, nota plasmid. Trends Microbiol..

[121] Fournes F., Val M.E., Skovgaard O., Mazel D. (2018). Replicate once per cell cycle: Replication control of secondary chromosomes. Front. Microbiol..

[122] Hülter N., Ilhan J., Wein T., Kadibalban A.S., Hammerschmidt K., Dagan T. (2017). An evolutionary perspective on plasmid lifestyle modes. Curr. Opin..

[123] Rivière C., Danos O., Douar A. (2006). Long-Term expression and repeated administration of AAV type 1, 2 and 5 vectors in skeletal muscle of immunocompetent adult mice. Gene Ther..

[124] Rivera V.M., Gao G., Grant R.L., Schnell M.A., Zoltick P.W., Rozamus L.W., Clackson T., Wilson J.M. (2005). Long-Term pharmacologically regulated expression of erythropoietin in primates following AAV-mediated gene transfer. Blood.

[125] Penaud-Budloo M., Le Guiner C., Nowrouzi A., Toromanoff A., Chérel Y., Chenuaud P., Schmidt M., Von Kalle C., Rolling F., Moullier P. (2008). Adeno-Associated virus vector genomes persist as episomal chromatin in primate muscle. J. Virol..

[126] Igoucheva O., Alexeev V., Yoon K. (2006). Differential cellular responses to exogenous DNA in mammalian cells and its effect on oligonucleotide-directed gene modification. Gene Ther..

[127] Bai H., Lester G.M.S., Petishnok L.C., Dean D.A. (2017). Cytoplasmic transport and nuclear import of plasmid DNA. Biosci. Rep..

[128] Lukacs G.L., Haggie P., Seksek O., Lechardeur D., Freedman N., Verkman A.S. (2000). Size-Dependent DNA mobility in cytoplasm and nucleus. J. Biol. Chem..

[129] Dauty E., Verkman A.S. (2005). Actin cytoskeleton as the principal determinant of size-dependent DNA mobility in cytoplasm: A new barrier for non-viral gene delivery. J. Biol. Chem..

[130] Stamenović D., Wang N. (2011). Stress transmission within the cell. Compr. Physiol..

[131] Zabner J., Fasbender A.J., Moninger T., Poellinger K.A., Welsh M.J. (1995). Cellular and molecular barriers to gene transfer by a cationic lipid. J. Biol. Chem..

[132] Tranchant I., Thompson B., Nicolazzi C., Mignet N., Scherman D. (2004). Physicochemical optimisation of plasmid delivery with cationic lipids. J. Gene Med..

[133] Ewert K., Ahmad A., Evans H.M., Schmidt H.-W., Safinya C.R. (2002). Efficient synthesis and Cell- Transfection properties of a new multivalent cationic lipid for non-viral gene delivery. J. Med. Chem..

[134] Patra J.K., Das G., Fraceto L.F., Campos E.V.R., Rodriguez-Torres M.D.P., Torres L.S.A., Torres L.A.D., Grillo R., Swamy M.K., Sharma S. (2018). Nano based drug delivery systems: Recent developments and future prospects. J. Nanobiotechnol..

[135] Sato Y.T., Umezaki K., Sawada S., Mukai S.-A., Sasaki Y., Harada N., Shiku H., Akiyoshi K. (2016). Engineering hybrid exosomes by membrane fusion with liposomes. Sci. Rep..

[136] Lin Y., Wu J., Gu W., Huang Y., Tong Z., Huang L., Tan J. (2018). Exosome-Liposome hybrid nanoparticles deliver CRISPR/Cas9 system in MSCs. Adv. Sci..

[137] Elsana H., Olusanya T.O.B., Carr-Wilkinson J., Darby S., Faheem A., Elkordy A.A. (2019). Evaluation of novel cationic gene based liposomes with cyclodextrin prepared by thin film hydration and microfluidic systems. Sci. Rep..

[138] Chandrawati R., Caruso F. (2012). Biomimetic liposome- and polymersome-based multicompartmentalized assemblies. Langmuir.

[139] Kotla N.G., Chandrasekar B., Rooney P., Sivaraman G., Larrañaga A., Krishna K.V., Pandit A., A Rochev Y. (2017). Biomimetic lipid-based nanosystems for enhanced dermal delivery of drugs and bioactive agents. ACS Biomater. Sci. Eng..

[140] Alavi M., Karimi N., Safaei M. (2017). Application of various types of liposomes in drug delivery systems. Adv. Pharm. Bull..

[141] Jesorka A., Orwar O. (2008). Liposomes: Technologies and Analytical Applications. Annu. Rev. Anal. Chem..

[142] Walker S.A., Kennedy M.T., Zasadzinski J.A. (1997). Encapsulation of bilayer vesicles by self-assembly. Nature.

[143] Walde P., Ichikawa S. (2001). Enzymes inside Lipid Vesicles: Preparation, reactivity and applications. Biomol. Eng..

[144] Torchilin V.P. (2005). Recent advances with liposomes as pharmaceutical carriers. Nat. Rev. Drug Discov..

[145] Stano P., Carrara P., Kuruma Y., Souza T.P., Luisi P.L. (2011). Compartmentalized reactions as a case of soft-matter biotechnology: Synthesis of proteins and nucleic acids inside lipid vesicles. J. Mater. Chem..

[146] Maina J.W., Richardson J.J., Chandrawati R., Kempe K., van Koeverden M.P., Caruso F. (2015). Capsosomes as long-term delivery vehicles for protein therapeutics. Langmuir.

[147] Nagahara A.H., Tuszynski M.H. (2011). Potential therapeutic uses of BDNF in neurological and psychiatric disorders. Nat. Rev. Drug Discov..

[148] Nagahara A.H., Merrill D., Coppola G., Tsukada S., Schroeder B.E., Shaked G.M., Wang L., Blesch A., Kim A., Conner J.M. (2009). Neuroprotective effects of brain-derived neurotrophic factor in rodent and primate models of Alzheimer’s disease. Nat. Med..

[149] Fumagalli F., Racagni G., Riva M.A. (2006). Shedding light into the role of BDNF in the pharmacotherapy of Parkinson’s disease. Pharmacogenomics J..

